# Review of available national guidelines for obstetric anal sphincter injury

**DOI:** 10.1007/s00192-020-04464-5

**Published:** 2020-08-13

**Authors:** Joanna C. Roper, Nirmala Amber, Osanna Yee Ki Wan, Abdul H. Sultan, Ranee Thakar

**Affiliations:** 1 Obstetrics and Gynaecology department, Croydon University Hospital, London Road, Croydon, CR7 7YE UK; 2 KIMS Hospitals, Secunderabad, India; 3 Department of Obstetrics and Gynaecology, Prince of Wales Hospital, The Chinese University of Hong Kong, Hong Kong, SAR Hong Kong; 4 St George’s University of London, London, UK

**Keywords:** AGREE II, Guidelines., Obstetric anal sphincter injury, Recommendations

## Abstract

**Introduction and hypothesis:**

Obstetric anal sphincter injuries (OASIs) are the most severe form of perineal trauma with potentially devastating effects on a mother’s quality of life. There are various national guidelines available for their management. The aim of this study was to review and compare recommendations from published national guidelines regarding management and prevention of OASI.

**Methods:**

We searched the PUBMED, EMBASE, MEDLINE, CINAHL and COCHRANE databases from January 2008 till October 2019 using relevant Medical Subject Headings (MeSH), including all subheadings. The guideline characteristics were mapped and methodological quality assessed with the Appraisal of Guidelines for Research and Evaluation (AGREE) II tool by three independent reviewers. To compare the methodological quality of the guidelines, the interpretation of the six domain scores were taken into consideration. By consensus of the authors, a score of 70% was taken as a cut-off, and scores above this were considered ‘high quality’.

**Results:**

Thirteen national guidelines on perineal trauma were included and analysed. Nine of these were specific to OASI. There is wide variation in methodological quality and evidence used for recommendations. AGREE scores for overall guideline assessment were > 70% in eight of the guidelines, with Australia-Queensland, Canada, the UK and USA scoring highest.

**Conclusions:**

The wide variation in methodological quality and evidence used for recommendations suggests that there is a need for an agreed international guideline. This will enable healthcare practitioners to follow the same recommendations, with the most recent evidence, and provide evidence-based care to all women globally.

## Introduction

Approximately 90% of females suffer from some degree of perineal tear during vaginal birth [1]. Obstetric anal sphincter injuries (OASIs) are associated with significant maternal morbidity including perineal pain, sexual dysfunction, and anal and urinary incontinence, which may persist for years after childbirth [2]. Complications of severe perineal tears include abscess formation, wound breakdown and rectovaginal fistulae. The number of complications is likely to be higher when poorly managed.

On a global front, the rates of OASI vary greatly. A systematic review which explored the rates of birth-related perineal trauma in low and middle-income countries showed that data were not available for most countries. The Philippines had the highest reported rate of 15%, with the lowest rate (0.1%) in Cambodia [3]. Similarly, the rates in the more developed Organization for Economic Co-operation and Development (OECD) countries vary from 3.1% in Canada to 0.2% in Poland [4]. This highlights the difficulty with accurate diagnosis of OASI, which underpins the ultimate management and outcome.

Guidelines are defined by the Institute of Medicine as ‘statements that include recommendations intended to optimise patient care, that are informed by a systematic review of evidence and an assessment of the benefits and harms of alternative care options’ [5].

The Appraisal of Guidelines for Research and Evaluation (AGREE) II instrument is the most commonly used, validated guideline appraisal tool [6]. Although there has been an overall increase in the quality of clinical guidelines over time, quality scores assessed with the AGREE instrument have remained moderate to low [7]. This finding is an impetus to guideline developers towards improving quality.

Few countries that have their own national guidelines on OASI. There are discrepancies and variations within each guideline, which leads to variation in obstetric practice in relation to protection of the perineum, type and frequency of episiotomy and management of OASI [3].

The aim of this study was to assess the methodological rigour of guideline development using AGREE II and compare recommendations from published national guidelines regarding management and prevention of OASI.

## Materials and methods

A search was performed in PUBMED, EMBASE, MEDLINE, CINAHL and COCHRANE databases from January 2008 till October 2019 using relevant Medical Subject Headings (MeSH) including all subheadings. Keyword search included the following: labour complications, anal canal, anal sphincter musculature, anal sphincter injuries, third degree, fourth degree, perineum, tear, laceration, disruption, rupture, trauma, disorder, incontinence, faecal, anal sphincter repair, suturing methods, end to end, overlap technique, recurrence and pregnancy after OASIS. In addition, web search engines, such as Google, were used to search for guidelines related to perineal tears and OASI.

All results were reviewed, guidelines extracted and duplicates removed. National guidelines on practice related to obstetric perineal trauma, in particular OASI, were included. Guidelines from individual hospitals were excluded. The guidelines in non-English languages (Dutch, Danish and Spanish) were translated into English using Google Translate.

### Guideline characteristics

Independent reviewers (NA and JR) recorded guideline characteristics including: country of origin, year of publication, principle developers, stakeholders involved, scope, consensus method, search databases, search period, endorsements and quality assessment methods.

### Assessment and analysis of methodological quality

Four reviewers (NP, GV, OW, JR) underwent training in the use of quality assessment using the AGREE II tool from www.agreetrust.org.

The AGREE assessment is divided into 23 core items and 2 overall assessment items. The core items are split into six domains of practice guideline quality: scope and purpose, stakeholders involvement, rigour of development, clarity of presentation, applicability and editorial independence. Each item is assessed on a 7-point Likert scale. Domain scores were calculated as recommended (Table 3).

Guidelines were independently scored using the AGREE assessment by three reviewers. By consensus of the authors, a score of 70% was taken as a cut-off, and scores above this were considered ‘high quality’. The intra-class correlation coefficient values were calculated using SPSS version 26.0.0.0. for all six domains to look at intra-rater agreement.

### Recommendations for clinical practice

Key recommendations were mapped using the following headings: classification, prediction and prevention, identification of OASIS, repair of OASIS, choice of suture materials, surgical competence, postoperative management and future deliveries. The recommendations, which were selected by an expert author from the RCOG guideline [8], were classified as ‘included and recommended’, ‘included with restriction on recommendation’, ‘insufficient evidence to support recommendation’ or ‘not mentioned’. References supporting clinical evidence were also mapped for comparison.

## Results

### Guideline search and selection (Fig. 1)

A literature search identified 2240 articles. Two guidelines [9, 10] were found through the search on www.google.com and 13 national guidelines were included. The guidelines for Queensland and South Australia were included because the geographical area covered is much larger than some of the other countries included. Moreover, no guideline covering the whole of Australia was identified. Two guidelines from Denmark were included because they cover different aspects of OASI care.

**Fig. 1:**
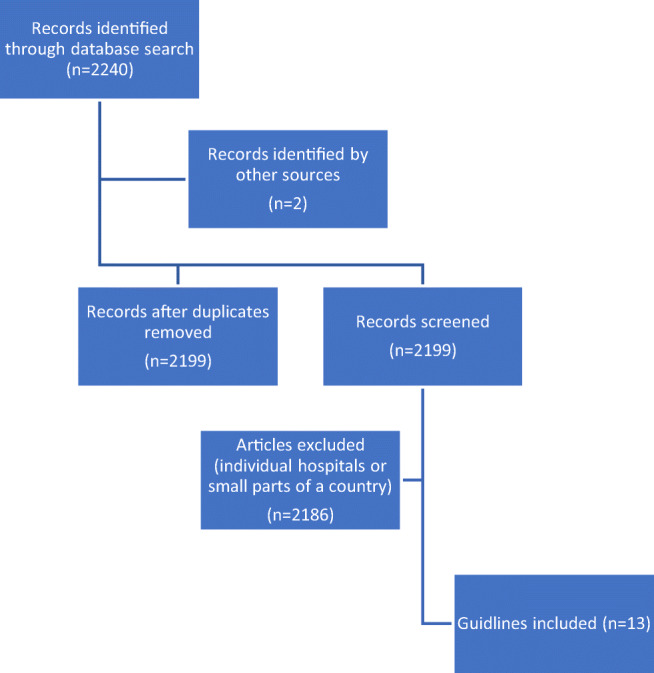
Flow diagram of selection of guidelines

### Guideline characteristics (Tables 1 and 2)

All guidelines identified obstetrics and gynaecology doctors involved in their development. One had a comprehensive team of developers, including women who had suffered OASIs [13]. Six had midwives as part of their development team. One [18] involved a coloproctologist.

**Table 1:** Guidelines included in this review

Name of guideline	Country of origin	Publication date
Government of South Australia- South Australian Perinatal Practice guideline. Third- and fourth-degree tear management	Australia	June 2018
Queensland Clinical Guidelines- Perineal care	Australia	June 2018
Austria Urogynaecology Working Group- Guidelines for the management of third- and fourth-degree tears after vaginal birth	Austria	2013
Society of Obstetrics and Gynaecology of Canada- Clinical Practice Guideline, Number 330. OASIS: prevention, recognition and repair	Canada	December 2015
Forebyggelse af sphincterruptur (Prevention of sphincter rupture)	Denmark	December 2015
Sphincterruptur: Diagnostik, behandling og opfolgning (OASI: Diagnosis, treatment and follow-up)	Denmark	2019
German Society of Gynaecology and Obstetrics - Management of 3rd- and 4th-degree tears after vaginal birth	Germany	October 2014
Institute of Obstetricians and Gynaecologists- Clinical Practice Guideline- Management of OASIS	Ireland	April 2014
Prevencion, diagnostico y tratamiento de episiotomia complicada (Prevention, diagnosis and treatment of complicated episiotomy)	Mexico	2014
Dutch Society of Obstetrics and Gynaecology: Risk factors for and interventions that reduce the risk of a total rupture during childbirth	The Netherlands	May 2013
Saudi Society of Obstetrics and Gynaecology, Policy and Procedure-PERINEAL TRAUMA	Saudi Arabia	July 2016
Royal College of Obstetrics and Gynaecology -Green-top Guideline No 29 Management of third- and fourth-degree perineal tears	UK	June 2015
American College of Obstetrics and Gynaecology- Practice Bulletin Number 198. Prevention and management of obstetric lacerations at vaginal delivery	USA	September 2018

**Table 2:** Characteristics of each guideline, including scope and developers

	Developers/stakeholders	Scope	Consensus	Search Database (evidence)	Search Period	Reviewed/Endorsed by	Quality assessment
Australia- South	3 Doctors 2 others	C E RF D R A F S	–	–		SA Health Safety and Quality Strategic Governance Committee	
Australia- Queensland	1 Obstetrics and gynaecology doctor 1 midwife	C E RF P D R A F S (FGM)	–	–	–	–	Queensland Clinical Guidelines Steering Committee Statewide Maternity and Neonatal Clinical Network (Queensland)
Austria	10 Obstetrics and gynaecology doctors 1 surgeon 1 midwife	C E RF P D R F S	DELPHI	–	Up to 30 Nov 2011	–	–
Canada	2 Doctors	C E RF P D R A F S	–	Cochrane Medline Embase	May 2011	Clinical Practice-Obstetrics &Family physician advisory committees	Canadian Task Force on Preventive Health Care
Denmark- 2015	3 Trainee speciality doctors 2 midwives 4 residents 1 consultant 2 physicians	C E RF P D	–	Cochrane Pubmed	Upto Nov 2014	2 × Midwife (1 associate professor), Clinical Associate Professor	–
Denmark- 2019	1 Midwife 1 physio 1 gastroenterology surgeon 13 others (assumed obstetrics and gynaecology doctors)	C E RF D R A F S	–	Cochrane Up-to-date Medline Pubmed	Depending on section. For updates to sections, searched from 2010 (prev version of guideline). Some sections no limit	Discussed at obstetrical guideline meeting, Annual meeting of Urogynaecological Society and Gynaecological Guideline meeting	Hierarchical classification of evidence for each recommendation
Germany	14 Urogynaecology doctors 1 midwife 1 surgeon (coloproctologist)	C E RF P D R F S	–	Pubmed Medline +Secondary literature	Austrian + Publications in English and German between Nov 2011 and Jan 2014	–	–
Ireland	2 Obs and gynae doctors	C E D R F S	–	Cochrane Medline Embase	June 2001-Dec 2011	3 Doctors, 1 GP, 2 × physiotherapist	–
Mexico	4 obs and gynae doctors 1 family medicine doctor	C RF P D R	–	Cochrane Pubmed	Last 5 years	2 Obstetrics and gynaecology doctors	Analysed hierarchical classification of evidence from each guideline used to formulate recommendations
The Netherlands	4 Gynaecologists 1 gastroenterologist 1 physio 1 midwife 1 radiologist 1 surgeon 1 epidemiologist Women with OASIs/increased risk of OASI	RF P D F S	–	Cochrane Medline Embase	Upto May 2013	1 × information specialist	AGREE II instrument
Saudi Arabia	2 Doctors	C RF P D	–	–	June 2016	1 × doctor	–
UK	5 Obstetrics and gynaecology or urogynaecology doctors	C E RF P D R F S	–	Cochrane Medline Embase	2006–2014	4 Obstetrics and gynaecology doctors	Clinical Governance Advice No. 1 Development of Green-Top Guidelines
USA	ACOG Committee on Practice Bulletins—Obstetrics in collaboration with 2 doctors	C E RF P D R F S	–	Cochrane Medline Internal resources and documents	Jan 1985-March 2018	–	US Preventive Services Task Force

**Index**

**-** = not reported, C = classification, E = epidemiology, RF = risk factors, P = preventative measures, D = diagnosis, R = repair, A = analgesia, F = follow-up**,**

S = subsequent birth

Guidelines were published between 2013 and 2019. There was variation in the scope. All included recommendations for the management of perineal tears sustained during childbirth. Some [13–15] were more focused on aspects such as prevention and risk factors for OASIs. All guidelines, except one, included the Sultan classification [8]. Twelve included a description of risk factors for perineal tears and ten made recommendations for prevention of tears.

Search strategy was described by nine [8, 9, 11, 13, 14, 16–18, 10]. The recommendations were based on research evidence in all of the guidelines, but the consensus method was only mentioned by one [12]. The quality assessment of the research evidence was described by seven, with one [13] using the AGREE II tool. The guideline development team members who reviewed or endorsed the recommendations and summary statements were not reported in four guidelines [12, 16, 18, 19].

### AGREE II scores (Table 3)

Eight guidelines had an overall score > 70%. Considering the domain scores the Dutch [13] and Irish [11] had the highest number scoring of > 70%, with five out of the six. Ten guidelines [8–11, 13, 14, 16–19] were assessed as ‘high quality’ in the ‘Rigour of development’ domain, which is the most valued domain for appraisal. ‘Clarity of presentation’ scored highly in all except one guideline [14] and therefore is the strongest domain across all the guidelines reviewed.

**Table 3:** Scores in each domain using the AGREE II tool

Domain	AGREE-II scaled domain score													
	AUSTRALIA-S	AUSTRALIA-Q	AUSTRIA	CANADA	DENMARK 2015	DENMARK 2019	GERMANY	IRELAND	MEXICO	NETHERLANDS	SAUDI ARABIA	UK	USA	Mean domain score
Scope and purpose	40 (57%)	60 (94%)	53 (81%)	63 (100%)	57 (89%)	40 (57%)	60 (94%)	56 (87%)	60 (94%)	56 (87%)	51 (78%)	56 (87%)	59 (93%)	84.5%
Stakeholder involvement	30 (39%)	42 (61%)	42 (61%)	39 (55%)	44 (65%)	22 (24%)	43 (63%)	49 (74%)	34 (46%)	55 (85%)	40 (57%)	28 (35%)	24 (28%)	53.3%
Rigour of development	84 (42%)	152 (89%)	118 (65%)	154 (90%)	165 (98%)	144 (83%)	151 (88%)	131 (74%)	131 (74%)	137 (78%)	79 (38%)	152 (89%)	126 (71%)	75.3%
Clarity of presentation	58 (91%)	61 (96%)	49 (74%)	61 (96%)	43 (63%)	58 (91%)	55 (85%)	53 (81%)	57 (89%)	52 (80%)	51 (78%)	57 (89%)	61 (96%)	85.3%
Applicability	35 (32%)	63 (71%)	20 (11%)	62 (69%)	44 (44%)	26 (19%)	59 (65%)	64 (72%)	23 (15%)	56 (61%)	42 (42%)	65 (74%)	42 (44%)	47.6%
Editorial independence	12 (17%)	25 (53%)	42 (100%)	23 (47%)	28 (61%)	13 (19%)	41 (97%)	27 (58%)	28 (61%)	34 (78%)	12 (17%)	24 (50%)	22 (44%)	54%
Overall guideline assessment	61%	94%	61%	94%	67%	78%	78%	67%	72%	78%	56%	94%	94%	76.5%
Recommendation	Ym	Y	Ym	Y	Ym	Y	Y	Ym	Y	Y	Ym	Y	Y	

Obtained score (percentage)

Domain scores were calculated by the following formula (obtained score – minimum possible score)/(maximum possible score – minimum possible score)

Y = recommended, Ym = recommended with modification

The intra-class correlation coefficient values for all AGREE II domains ranged from 0.62 to 0.94 (scope and purpose: 0.81; stakeholder involvement 0.94; rigour of development 0.79; clarity of presentation: 0.62; applicability: 0.88; editorial independence: 0.92). This indicated a high level of intra-rater agreement in all except ‘Clarity of presentation’.

### Recommendations for practice (Table 4)

Key recommendations were mapped.

1. Classification of perineal trauma was presented in 12.
2. Definition of a rectal button hole tears was mentioned in six.
3. Risk factors for OASI were discussed in 12.
4. Prevention of OASI with perineal protection was recommended in four, while five said there was insufficient evidence to recommend it.
5. Prevention of OASI during instrumental delivery using a mediolateral episiotomy was recommended in seven. One reported there was insufficient evidence to recommend episiotomy for all instrumental deliveries. The German and Danish guidelines only mentioned episiotomy with vacuum delivery, while forceps delivery was not mentioned.
6. Prevention of OASI by using a warm compresses on the perineum in labour was recommended by six. Two suggested insufficient research to recommend it.
7. Diagnosis of perineal trauma with digital rectal examination was recommended in seven. Four stated that rectal examination was only necessary in some circumstances.
8. Repair of OASI in theatre with general or regional anaesthesia was recommended in eight. South Australia recommended that only 3b/c and fourth-degree tears require this, whereas 3a tears can be repaired in the delivery room [20].
9. Nine recommended that the person conducting the OASI repair should be adequately trained.
10. Five made specific recommendations about the repair for the anal mucosa.
11. Separate repair of the internal anal sphincter (IAS) from the external anal sphincter (EAS) was recommended in eight. The guideline from the USA stated if the IAS can be identified it can be repaired separately or ‘as part of the distal portion of the reinforcing second layer of the rectal muscularis’, while The Netherlands mentioned this was only needed if it was possible without further exploration of the wound.
12. Repairing a full-thickness EAS tear with overlapping or end-to-end technique was recommended in 11.
13. Recommendations about suture type for repair were mentioned in ten.
14. Rectal examination after completion of the repair was recommended in four.
15. Broad-spectrum antibiotic prophylaxis at the time of repair was recommended by ten.
16. A post-operative course of prophylactic antibiotics was recommended by three. Queensland recommended this for fourth-degree tears only and advised to consider it in third-degree tears. Three said there was insufficient evidence to recommend any antibiotics following repair.
17. Post-operative follow-up with physiotherapy was recommended by eight. Canada stated that this was only necessary for women with anal incontinence.
18. A post-operative course of laxatives was recommended by ten.
19. A risk of repeat OASI in a subsequent pregnancy was described in nine.
20. Follow-up with endo-anal ultrasound was recommended in five.
21. Episiotomy in a subsequent delivery was mentioned in six, but the lack of sufficient evidence to recommend it was discussed.
22. Caesarean section was recommended for women who were symptomatic of anal incontinence in a subsequent pregnancy in nine.

**Table 4:** Summary of recommendations for OASI

	Australia- South	Australia- Queensland	Austria	Canada	Denmark 2015	Denmark 2019	Germany	Ireland	Mexico	The Netherlands	Saudi Arabia	UK	USA
Classification of perineal trauma	✓	✓	✓	✓	✓	✓	✓	✓	✓	N	✓	✓	✓
Buttonhole tears	✓	✓	N	✓	N	N	✓	N	N	N	✓	✓	N
Risk factors for OASI	✓	✓	✓	✓	✓	✓	✓	N	✓	✓	✓	✓	✓
Prevenetion- perineal protection	N	i	i	i	✓	N	i	N	N	✓	✓	✓	i
Prevention- mediolateral episiotomy for instrumental delivery	N	✓	N	✓	✓	(✓)	(✓)	N	✓	✓	✓	✓	i
Prevention- warm compress	N	✓	N	✓	✓	N	i	N	N	i	✓	✓	✓
Diagnosis- examination with digital rectal examination	(✓)	✓	✓	(✓)	N	✓	(✓)	✓	N	✓	✓	✓	(✓)
Repair- in theatre with regional analgesia	(✓)	✓	✓	✓	N	✓	✓	✓	N	✓	N	✓	N
Repair- trained person doing repair	✓	✓	✓	✓	N	✓	✓	✓	N	✓	N	✓	N
Repair – mucosa continuous or interrupted, position of knots	N	✓	N	✓	N	✓	N	N	N	N	N	✓	✓
Repair- IAS separately if torn	✓	✓	✓	✓	N	✓	✓	✓	N	(✓)	N	✓	(✓)
Repair- full thickness EAS end to end or overlap	✓	✓	✓	✓	N	✓	✓	✓	✓	✓	N	✓	✓
Suture materials	✓	✓	✓	✓	N	✓	✓	✓	✓	N	N	✓	✓
Post repair PR	✓	N	✓	✓	N	N	N	N	N	N	N	✓	N
Broadspectrum antibiotics at time of repair	✓	✓	✓	✓	N	✓	✓	✓	✓	N	N	✓	✓
Post repair antibiotics	✓	(✓)	✓	N	N	i	✓	i	N	N	N	i	N
Recommend physiotherapy	N	✓	✓	(✓)	N	✓	✓	✓	N	✓	N	✓	✓
Post repair use of laxatives	✓	✓	✓	✓	N	✓	✓	✓	✓	N	N	✓	✓
Risk of OASI recurrence quoted	N	✓	✓	✓	N	✓	✓	✓	N	✓	N	✓	✓
Follow-up endoanal ultrasound recommended	N	✓	N	N	N	N	✓	✓	N	(✓)	N	✓	N
Episiotomy at subsequent delivery	i	i	I	N	N	N	i	N	N	I	N	i	N
Advise caesarean section for symptomatic women in subsequent pregnancy	✓	✓	✓	✓	N	✓	✓	✓	N	N	N	✓	✓

✓ = included and recommended, (✓) = recommended with restrictions, i = insufficient evidence for recommendation, N = not mentioned

## Discussion

Although there was a wide variation in methodological quality and evidence used for recommendations, all guidelines scored high quality (> 70%) in at least one domain of the AGREE II tool. Eight were high quality ‘Overall Guideline Assessment’ scores and therefore were classed as ‘Recommended’. Four guidelines scored > 90% overall [8, 16, 17, 19]. Interestingly, some of the individual domain scores for these were quite low.

Clinical practice guidelines are typically developed by a group of experts and healthcare professionals in the subject. Guidelines that have a small or restricted development team could introduce bias to the recommendations made. The AGREE II tool suggests that guideline development should include individuals from the relevant professional groups and also consult the patient groups affected by the guideline. On review of the development groups, a range of professionals were involved. Some consisted of doctors alone, while others included midwives, physiotherapists, colorectal surgeons and radiologists. The Dutch development team were the only group involving women who had experienced OASI and therefore scored the highest (85%) in ‘Stakeholder involvement’. Denmark (2019) and USA obtained the two lowest scores (24% and 28% respectively). This is because the guideline development team were not from a wide range of specialities, and the views of the target population had not been taken into consideration. It could be argued that all the stakeholders who would be using the recommendations from a guideline should be involved, whilst the patient population should form a vital part of the development team.

All guidelines, apart from one [14], scored highly (range 63%–96%) in ‘Clarity of presentation’. It is important that the content of the guideline is user-friendly. In this domain, the tool assesses how specific the recommendations are, that the options for management are clear, and whether key recommendations are easily identifiable. This ensures users can quickly identify the information they are looking for. The recommendations made need to be readily accessible, easy to interpret and applicable to a wide range of patients and scenarios.

There was variation in the scope of the guidelines. This is understandable since the titles imply a variation in the content. Some are more general, looking at perineal injuries as a whole [15, 16, 19, 9], and others are more focused on OASIs [8, 10–14, 17, 18, 20]. Whilst scope and content may vary, it is important that this is described in the outline of the guideline. The title of the guideline should be precise to increase its clinical applicability, therefore making it accessible and easily found when required in clinical practice. Ambiguity in a title, for example, ‘Perineal care’ or ‘Perineal trauma’ [15, 19], may lead to clinicians not accessing the guideline because the content is not clear. By contrast, ‘OASIS: prevention, recognition and repair’ [17] is self-explanatory.

The Saudi guideline had the lowest overall guideline assessment (56%). ‘Editorial independence’ (17%) and ‘Rigour of development’ (38%) were also low. When developing a guideline, it is essential to research the available evidence on the subject in order to make up-to-date and relevant recommendations. This may have been done thoroughly but the evidence for recommendations was not well documented. A clear explanation for the recommendations could help the users to weigh the level of evidence used and apply them clinically. It is also important for the user to have knowledge of the evidence used so that they can check for updates and follow accordingly. ‘Editorial independence’ analyses whether any funding bodies or conflicts of interest could have influenced the developers. Variations in the methods used to identify and assess the evidence result in variations in recommendations. When individual recommendations are reviewed it is important to note the evidence quoted and how it is interpreted. For example, the German guideline quoted Aasshiem et al.’s systematic review for warm compresses on the perineum but concluded that it ‘cannot be conclusively evaluated on account of inadequate or contradictory data’. However, the review concludes that warm compresses on the perineum decrease the occurrence of perineal trauma [21].

Looking in more detail at the recommendations included and evidence used for each guideline the following observations were made:

### Classification

Classification of perineal trauma ensures a thorough evaluation of anatomy and therefore an accurate repair of the trauma. OASI missed at delivery can effect a woman’s physical and emotional recovery [22]. All guidelines, except the Dutch guideline [13], recommended classification with the widely accepted Sultan criteria [8]. This is probably because it was beyond the scope of the guideline as its title, ‘Risk factors for and interventions that reduce the risk of a total rupture during childbirth’, implies that diagnosis of tears were not covered.

A rectal button hole tear is a tear of the anorectal mucosa and vagina with an intact anal sphincter which can lead to serious consequences such as a rectovaginal fistula [2]. It may present with an apparently intact perineum, making it more difficult to diagnose [2]. It is not part of the original classification and should not be labelled as a fourth-degree tear but is an important inclusion to be highlighted to clinicians. Only five of the guidelines mentioned button hole tears.

### Diagnosis of OASI

Without a digital rectal examination anorectal mucosal injury cannot be excluded [8]. Seven of the guidelines recommended digital rectal examination after vaginal delivery to diagnose and classify perineal trauma. Four of the guidelines recommended rectal examination, but only in certain cases. For example, the Canadian guideline reports ‘including a rectal examination for those with a tear that is more than superficial in depth’ [17]. The South Australian guideline stated ‘for all episiotomies or if tear extending to anal verge’ [20]. Without a digital rectal examination perineal trauma, including button hole tears, can easily be missed and lead to severe consequences for women.

### Prediction and prevention

Consistent risk factors for OASI were mentioned in 12 of the guidelines but some differences were noted. Increasing birth weight and instrumental delivery were mentioned in all except the Irish guideline, probably because it was beyond their intended scope. Yet, risk factors are an important part of a guideline. They can help in counselling patients during pregnancy and delivery.

In recent years there has been an increasing interest in prevention of OASI. Prevention was mentioned in ten guidelines, to varying degrees. Warm compresses were mentioned in eight; however, two suggested insufficient evidence for this. The reference used was consistently the systematic review by Aasheim et al. in reducing perineal trauma [21].

Perineal protection was recommended in four. Five others mentioned that there were insufficient data to recommend it. This may be due to timing of production, given that some of the research recently completed may not have been available during guideline development. This highlights the time-limited nature of guidelines and the importance of reviewing them at a fixed interval.

Episiotomy for instrumental delivery was recommended in nine. Two of these (German and Danish (2019)) only referred to vacuum delivery, which may be because forceps are not popularly used in those countries. There is growing evidence that episiotomy is protective of OASI in the presence of an instrumental delivery and due consideration needs to be given to this.

### Repair of OASI, suture materials and surgical competence

A detailed description of repair technique for the sphincter is an essential component of a guideline on OASI. It is suggested that it is done in theatre with regional or general anaesthetic by a person who is trained in repair of OASI [2]. These recommendations were made by seven guidelines. Both Australian guidelines described repair by a trained person, but only certain cases to be done in theatre. These were described as ‘difficult or extensive trauma’ [19] or ‘all 3b and c and fourth degree tears, but only 3a without adequate analgesia’ [20].

Depending on the grade of tear, different tissues require repair. The anorectal mucosa can be repaired in a continuous or interrupted suture. The knots of the sutures can either be in the anal canal or within the tissue layers [8]. Five described the repair of anorectal mucosa in this way. There was variation in the recommendations for repair of the IAS. It is known that women who have persistant IAS defects are more likely to have symptoms than those who do not [8].

The recommendation for EAS repair seems to be more unified. Eleven guidelines included the option for repair of a completely torn EAS with overlap or end-to-end technique. Six of these used the Cochrane review by Fernando et al. as a reference [23]. This is probably because the level of evidence to support this is higher.

### Post-operative management

Upon completion of the repair, a second digital rectal examination is recommended to confirm complete repair and also to check sutures have not inadvertently been inserted through the anorectal mucosa [2]. This was recommended in four guidelines. Without inclusion a clinician may not be aware of this step, which can lead to serious complications. Sutures breaching the anorectal mucosa can lead to recto-vaginal fistula formation, which cause faecal and flatus incontinence and are particularly difficult to repair [2].

A Cochrane review on antibiotic prophylaxis during OASI repair identified only one randomized control trial (RCT) [24, 25]. Either the RCT or the Cochrane review were cited as a reference for prophylactic antibiotics at the time of repair by seven of the ten guidelines that recommended this. A Cochrane review is the highest quality of research evidence and therefore, if available, should be reviewed to make recommendations for practice.

There is no evidence for a course of post-operative antibiotics. Only three guidelines recommended this, using expert opinion as evidence; another three stated there was insufficient evidence. When there is a lack of evidence it is important that this is stated to ensure the user understands the rationale for recommendations made. An expert opinion can be very valid in this situation. It is also useful to identify the gaps in research and state them in a section for future research.

Other post-operative care that is mentioned are physiotherapy and laxatives. Ten guidelines recommended a course of laxatives during the post-operative period, seven of which used the same RCT evidence by Mahony et al. [26]. Provision of a laxative is an important step in the postoperative management of OASI as constipation can lead to impaction and a breakdown of the repair.

### Future deliveries

Subsequent pregnancy can pose a dilemma to mothers and clinicians, due to recurrence of tears and anorectal symptoms. Subsequent pregnancy was discussed in ten guidelines. Nine of them quoted a risk of recurrence, with a range of 3% to 8%.

Five guidelines advise investigations for women during the follow-up period, including endo-anal ultrasound. The use of a dedicated perineal clinic was recommended in three guidelines, recognizing limitation of resources.

Research in the role of elective episiotomy during a subsequent vaginal delivery following OASI has recently been published. D’Souza et al. concluded that mediolateral episiotomy in subsequent vaginal deliveries decreases the recurrence rate of OASI in women who have had a previous OASI by 80% [27]. Six guidelines commented on the lack of evidence in this area, probably as their development predated this publication.

It is important that the guidelines provide the level of, or lack of evidence, for each recommendation. Some guidelines used other clinical guidelines as a references; for example, the Queensland guideline [19] used the RCOG guideline [8] as a reference for evidence of repair of the EAS and recommendation of laxative use after OASI. This may be seen as an indirect way of reviewing the evidence available and therefore possibly lead to inaccurate recommendations. By only considering guidelines as a reference this may not represent inclusion of the most recent publications.

### Comparison to other studies: Nygaard and Tsakiridis

Nygaard et al. recently appraised national guidelines for management of obstetric perineal lacerations [28] using the AGREE II tool but also included other forms of perineal trauma. However, the current evaluation focuses on OASI guidelines and includes six other guidelines. It also maps recommendations based on inclusion and evidence. However, similar to our paper, they too found the quality of guidelines to be highly variable. Tsakiridis et al. reviewed three guidelines on OASI in a descriptive review including quality of evidence, but without appraisal using AGREE II [29].

### Strengths and limitation

The strengths of this study include its originality, search strategy, methodological design and inclusion of a variety of countries. The guidelines in non-English languages have been translated into English using Google Translate, which can lead to a bias that could be avoided with the help of a professional translator. There was reasonable agreement between the three reviewers with discrepancies resolved through discussion and reviewed by an expert (RT). All guidelines includeed were from high resource countries, which limits generalization to middle and low-resource countries. Since well-trained professionals rather than high financial resources are needed for prevention, diagnosis and treatment, the suggestion of an international guideline is pertinent.

It is not possible to make recommendations of guidelines based on our analysis because of the variety of scope and purpose. There are some that have more robust development and are easier to interpret than others. And there are some that appear to have little evidence for the recommendations made. We therefore recommend a coordinated approach to development of guidelines.

The users of OASIs guidelines should be aware of their development methods; guidelines developed without a standardized methodological process may lead to clinical inaccuracies. In particular, we urge clinicians to be aware of the date of publication, and the evidence that has been published since. We advocate clinicians to explore the strength of the evidence used as this may highly impact whether recommendations are appropriate for certain clinical scenarios. Furthermore, guidelines are created based on the dates the search was conducted and it may take years before the guideline is published, following the review and consultation process.

We recommend guideline developers use a standardized method, such as AGREE II, using the latest evidence, together with a specific development team from many areas of clinical practice, including patient representatives. Finally, guideline review must be carried out on a regular basis to ensure it is in line with current evidence. A single universal guideline could reduce variations in clinical practice.

### Conclusion

No national guideline in this review strictly followed the standardized approach to guideline development as described in AGREE II. Given the variation in development protocol and interpretation of evidence in forming recommendations, the findings in this study justify the critical appraisal of the national OASIs guidelines and use of a tool such as AGREE II. Clinicians are advised to use current guidelines exercising awareness of guideline development, timing and available evidence used.
